# Enhancement in external quantum efficiency of AlGaInP red μ-LED using chemical solution treatment process

**DOI:** 10.1038/s41598-021-83933-3

**Published:** 2021-02-25

**Authors:** Byung Oh Jung, Wonyong Lee, Jeomoh Kim, Myungshin Choi, Hui-Youn Shin, Minho Joo, Sukkoo Jung, Yoon-Ho Choi, Moon J. Kim

**Affiliations:** 1 Materials & Devices Advanced Research Institute, LG Electronics, LG Science Park, 10, Magokjungang 10-ro, Gangseo-gu, Seoul, 07796 South Korea; 2 Department of Materials Science and Engineering, The University of Texas at Dallas, 800 W Campbell Rd., Richardson, TX 75080 USA

**Keywords:** Materials science, Nanoscience and technology, Optics and photonics, Physics

## Abstract

To investigate the effects of their surface recovery and optical properties, extremely small sized (12 µm × 12 µm mesa area) red AlGaInP micro light emitting diodes ($\upmu$ LED) were fabricated using a diluted hydrofluoric acid (HF) surface etch treatment. After the chemical treatment, the external quantum efficiencies (EQEs) of $\upmu$-LED at low and high injection current regions have been improved by 35.48% and 12.86%, respectively. The different phenomena of EQEs have a complex relationship between the suppression of non-radiative recombination originating from the etching damage of the surface and the improvement of light extraction of the sidewalls. The constant enhancement of EQE at a high injection current it is attributed to the expansion of the active region’s sidewall surface area by the selective etching of AlInP layers. The improved EQE at a low injection current is related to the minimization of the surface recombination caused by plasma damage from the surface. High-resolution transmission electron microscopy (HR-TEM) revealed physical defects on the sidewall surface, such as plasma-induced lattice disorder and impurity contamination damage, were eliminated using chemical treatment. This study suggests that chemical surface treatment using diluted HF acid can be an effective method for enhancing the $\upmu$-LED performance.

## Introduction

**Figure 1:**
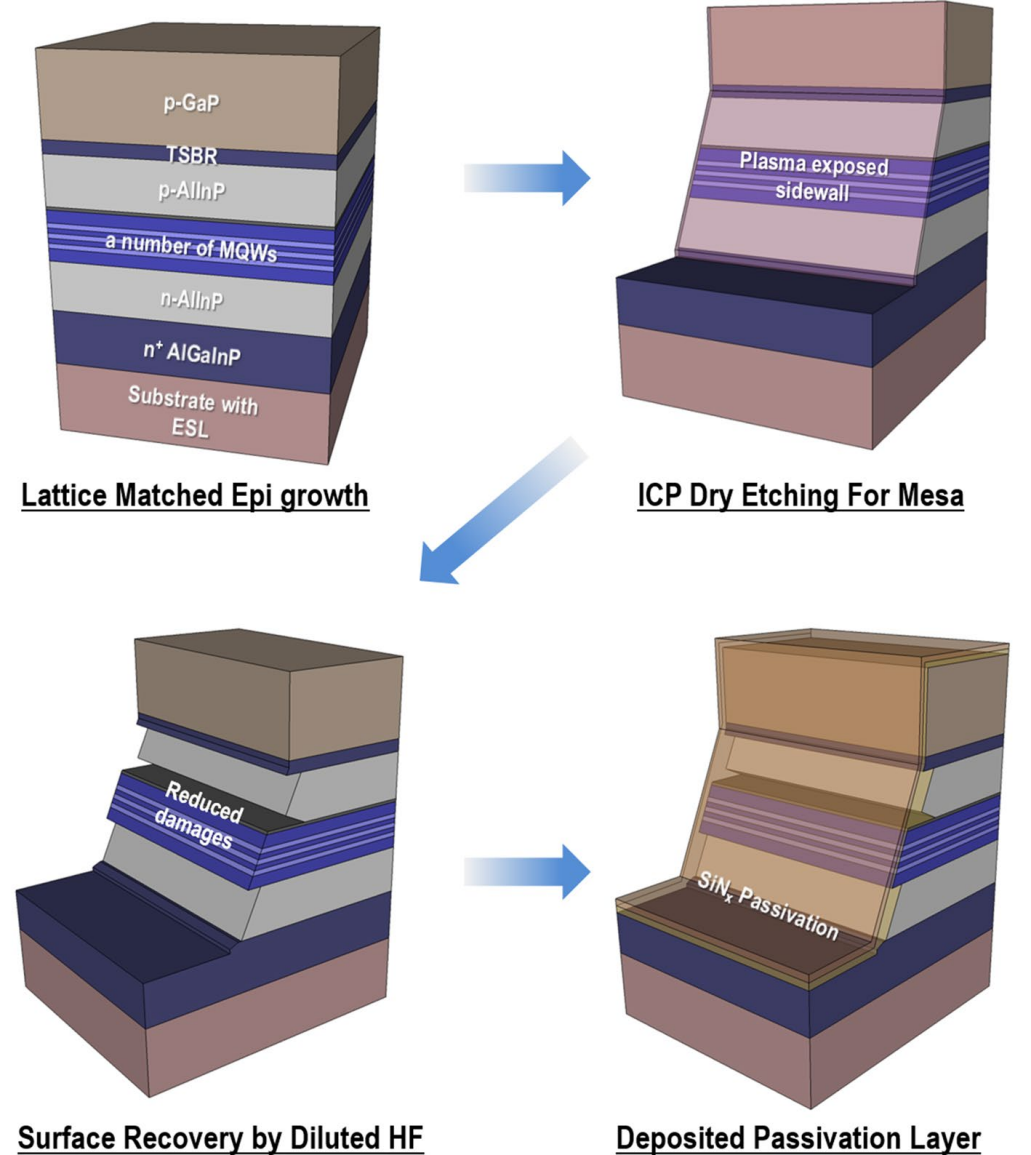
Schematic of AlGaInP-based μ-LED fabrication procedure. MOVPE growth of epilayers (upper-left panel), followed by the ICP dry etching process for mesa patterning (upper-right panel). Subsequently, the exposed sidewalls were treated using a diluted HF solution for surface recovery (bottom-left panel). The SiN_x_ layer was coated on mesa sidewalls to form passivation (bottom-right panel).

High-brightness RGB full-color light-emitting diodes (LEDs) with low energy consumption and high efficiency are required for display devices. Above all, mini LEDs (m-LEDs) and micro LEDs (μ-LEDs) have emerged as next-generation display applications in recent years^1,2^. For full-color display devices, blue and green LEDs based on III-nitride In_x_Ga_1−x_N ternary alloy systems have reached a sufficiently high emission efficiency^3^. However, the emission efficiency of the visible red region remains a major challenge owing to the difficulty of high indium content caused by the large lattice mismatch between GaN and InN. The (Al_x_Ga_1−x_)_y_In_1−y_P quaternary alloy systems are one of the most promising candidates for high-efficiency lighting sources of red emission better than III-nitride LEDs^4^. However, although the internal quantum efficiency (IQE) of AlGaInP quaternary based LEDs has reached nearly 100% through lattice-matched hetero-epitaxial growth, the relatively low light emission efficiency compared to blue and green LEDs remains a critical issue in preventing the high performance of AlGaInP-based LEDs^5^. Several approaches have been proposed to improve the light emission efficiency of AlGaInP-based LEDs through the various designs of the chip geometry^6^, wafer bonding technique^7^, surface texture with a patterned structure^8,9^, and self-assembled (or embedded) nano-architecture^10–12^. Their efforts mainly focus on the improvement of the light extraction efficiency (LEE) to enhance the external quantum efficiency (EQE) of the LEDs because it is difficult for photons to escape into the air from a semiconductor with a high-refractive-index. Despite these efforts, there is still a significant discrepancy between the EQE and IQE of red LEDs. Therefore, one of the key subjects for improving the EQE is increasing the escape of a large number of photons from the active regions of AlGaInP-based LEDs. There are other efforts to enhance the EQE in μ-LED research. Kou et al. reported that a smaller LED size can lead to a high Shockley–Read–Hall (SRH) non-radiative recombination at the sidewall defect sites caused by plasma dry etching originating from the higher surface-to-volume ratio^13^. Bulashevich et al. also reported the channel of carrier losses by surface recombination velocity (SRV) and carrier diffusion length. They found that the narrow bandgap and zinc blend crystal structure have relatively higher values than those of wurtzite crystal structures such as GaN and InGaN^14^. As a result, surface recombination has a detrimental influence on the efficiency of AlGaInP-based μ-LEDs owing to the relatively enhanced non-radiative recombination at the sidewalls of the active region as the chip size is reduced^15^. For these reasons, to achieve a high light emission efficiency, the red emission of μ-LEDs in particular must be obtained through a high LEE and minimized emission losses from the elimination of the surface recombination. In this report, we investigate the effect of the surface recovery on the efficiency of AlGaInP-based μ-LEDs by using a simple chemical etching process applying diluted hydrofluoric acid (HF). Figure 1 shows the key mesa fabrication processing steps for AlGaInP-based red LEDs. A conventional plasma dry etch process was conducted to form a mesa structure, followed by a diluted HF chemical solution treatment and deposition of a silicon nitride (SiN_x_) passivation layer. The diluted HF chemical treatment was found to not only remove the surface defects, such as the crystalline disorder and impurities on the sidewall surface of the active layer, coming from the plasma dry etching process, but also increase the sidewall area of the active region by selectively etching the AlInP cladding layers. As a result, the EQE of the red emission improved by over 30%.

## Experimental methods

A conventional metalorganic vapor-phase epitaxy (MOVPE) was carried out to grow AlGaInP-based epitaxial layers on a lattice-matched GaAs basal substrate. The epi structure of μ-LEDs consisted of a GaInP etching stop layer (ESL), *n*^+^-AlGaInP contact layer, *n*-cladding AlInP, AlGaInP active layers including a number of multiple quantum wells (MQWs), a thin AlInP layer with high Al content, *p*-cladding AlInP, AlGaInP tensile strain barrier reducing (TSBR) layer, magnesium-doped *p*-GaP windows layer, and *p*^+^-GaP:C ohmic contact layer. The *p*-mesa region was defined using an inductively coupled plasma-reactive ion etching (ICP-RIE) dry etching process with a photolithography patterning procedure to fabricate micro-sized LEDs. Subsequently, a diluted HF solution (DI:HF = 3:1) was applied to the fabricated device as a chemical treatment to eliminate the compromised area of the mesa sidewalls during the plasma etching process and selective etching of AlInP epilayers. An indium tin oxide (ITO) layer was then deposited using RF magnetron sputtering on the *p*-type GaP epitaxial layer as a current spreading and an ohmic contact layer. Finally, a thin dielectric SiN_x_ layer was deposited sequentially using plasma-enhanced chemical vapor deposition (PECVD) as a surface passivation layer. The surface morphology and microstructural characterization of AlGaInP-based μ-LEDs were carried out using Cs-corrected scanning transmission electron microscopy (Cs-corrected STEM, FEI Titan G2 60–300) operated at 300 kV. The compositional analysis of the mesa sidewalls of the active region was obtained from energy dispersive X-ray (EDX) spectroscopy in scanning TEM mode (FEI Talos F200X) operated at 200 kV. The samples were milled using a dual-beam focused ion beam (DB-FIB, FEI Scios 2 DualBeam) operated within the range of 5–30 kV.

## Results and discussion

**Figure 2:**
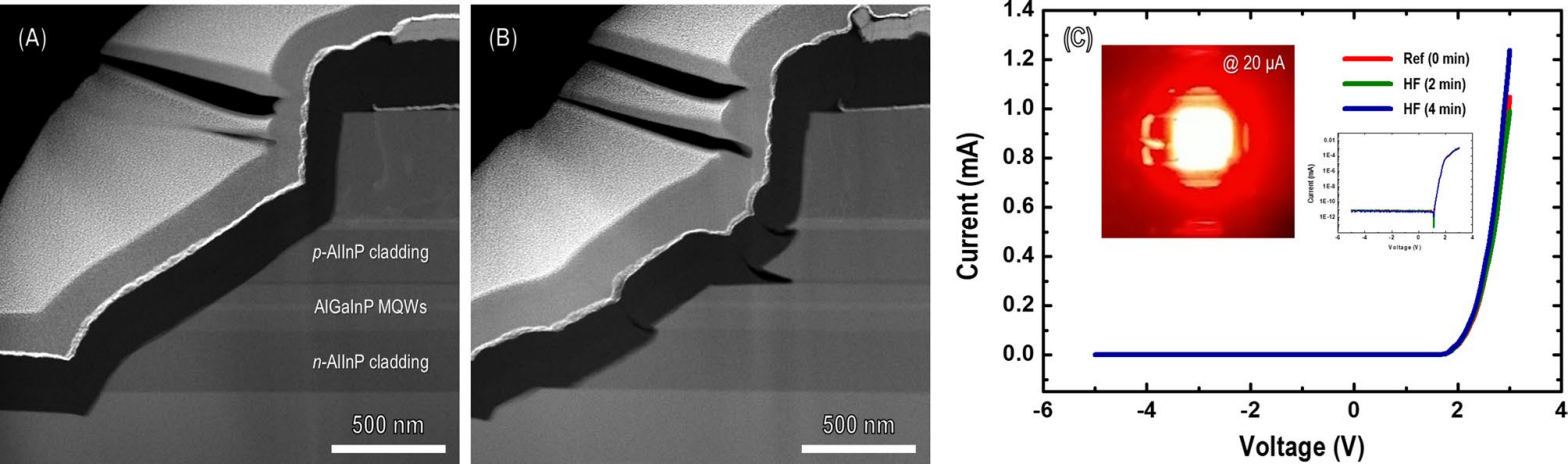
Low-magnification cross-sectional scanning TEM images of mesa sidewalls: (**A**) before diluted HF chemical treatment, (**B**) after diluted HF (4 min) treatment, (**C**) *I*–*V* characteristic curve of three different AlGaInP-based $\upmu$-LEDs as a function of applied bias voltage (the legend describes the diluted HF treatment time and Ref. indicates a treatment of 0 min). The inset shows light-emission photographs and *I*–*V* versus the log scale.

To explore the effect of the diluted HF chemical treatment on the mesa sidewalls of the μ-LED structure, the spatial distribution of the thickness and morphology of the upper and lower AlInP cladding layers on the AlGaInP active region were investigated using cross-sectional scanning TEM. Figure 2A,B show low-magnification high-angle annular dark-field (HAADF) STEM images of the mesa sidewall region. The HAADF image in Fig. 2A shows the typical mesa pattern formed on the sidewalls of the μ-LED. The etching depth and slant angle were measured to be approximately 1.3 μm and 60°, respectively. By contrast, a different sidewall morphology of a μ-LED is clearly shown in Fig. 2B. The two *n*- and *p*-AlInP cladding layers, including a thin high-Al-content AlInP layer above and below the active layers, were selectively etched using a diluted HF chemical solution compared with AlGaInP active layers. In general, the cladding layers related to AlInP contain a higher aluminum composition than the AlGaInP-based active region to manipulate the bandgap energy and strain. Although a diluted HF solution has been widely known as an etchant for oxide-related materials in the semiconductor industry, control of the HF concentration on the solution can determine the selective etching properties according to the aluminum composition of epilayers owing to their reactivity with aluminum^16^. This etching behavior depending on the aluminum content can play a vital role in enhancing the light emission efficiency in μ-LEDs, which will be discussed in detail in the next section of this article. The electrical characteristics of the μ-LEDs were investigated by measuring the current–voltage (*I*–*V*) characteristic curves to identify the effect of the selectively etched AlInP layers by the diluted HF chemical treatment. The inset in Fig. 2C shows the red emission electroluminescence (EL) image of μ-LED taken under 20 μA. Figure 2C shows the typical rectifying behavior of the μ-LEDs, with a turn-on voltage of ≈ 1.85 V regardless of the diluted HF chemical treatment. The injection currents of three sets of μ-LEDs using time frames of 0 min (Ref) and 2 and 4 min (diluted HF) at their turn-on voltages are 14,4, 12.8, and 15.6 μA, respectively. At an operating voltage of 3 V, the injection currents of Ref, 2 min, and 4 min were 1.05, 0.99, and 1.24 mA, respectively. Although there are differences in the injection currents between μ-LEDs at the same operating voltages, such small changes in injection current indicate that the diluted HF chemical treatment does not significantly affect the electrical characteristics of μ-LEDs.

**Figure 3:**
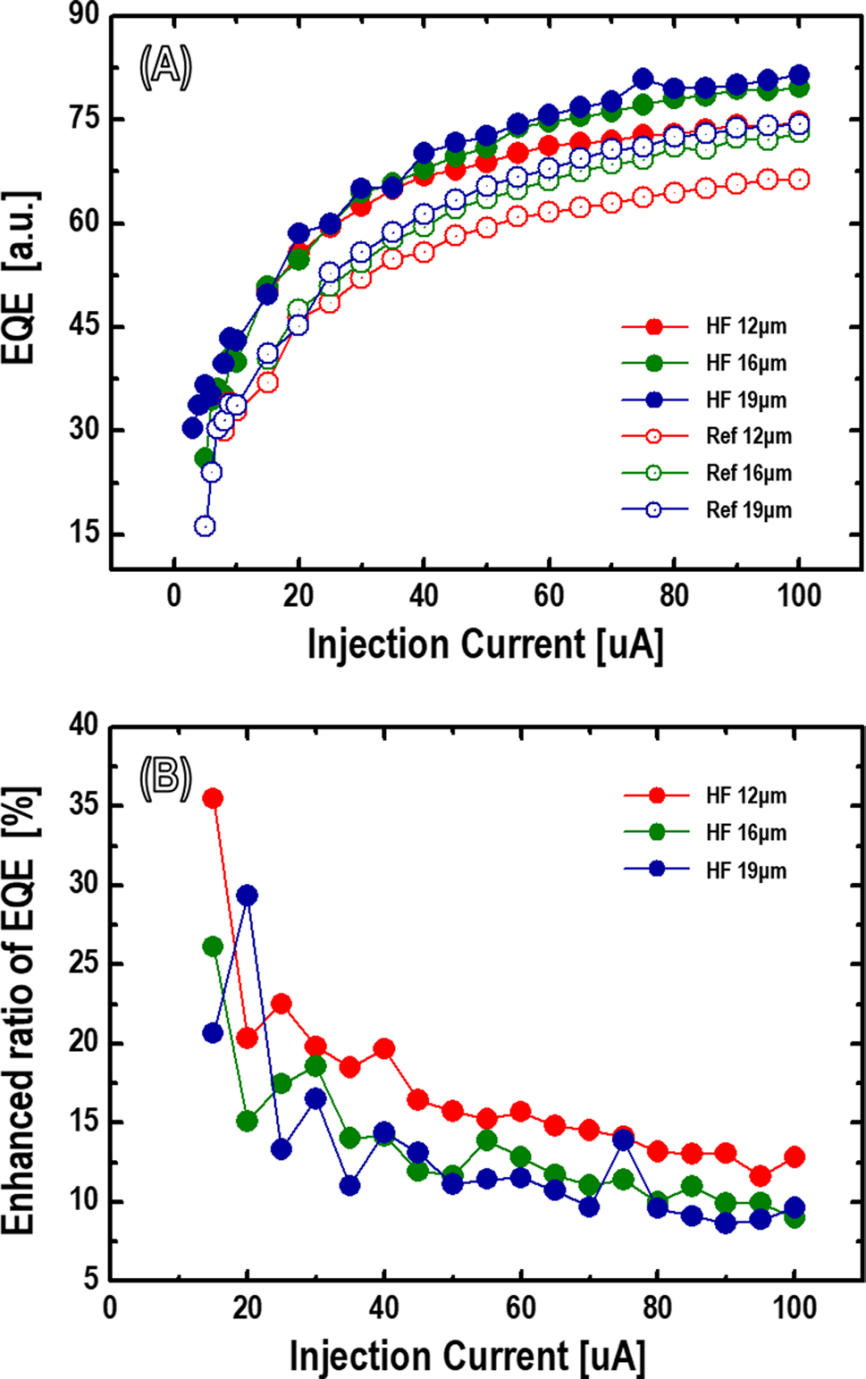
(**A**) Dependence of EQE on current injection (the legend describes the mesa size and presence or absence of the diluted HF chemical treatment), *a.u.* arbitrary units. (**B**) Enhanced ratio of EQEs versus injection current as a function of mesa size.

To further understand the influence of diluted HF chemical treatment, the optical characteristics of the diluted HF solution treatment were investigated by adopting the 4 min chemical treatment to the μ-LEDs with different mesa sizes. Figure 3A shows the EQE of μ-LEDs as a function of injection current according to the diluted HF chemical treatment process. The EQEs improved remarkably upon increasing the injection current from 3 to 100 μA. The measured EQEs show a typical size-dependent behavior regardless of the diluted HF chemical treatment. The EQE of the larger mesa size of μ-LED is higher than that of small mesa size, as described elsewhere in detail^17^. However, the EQEs of the μ-LEDs with diluted HF chemical treatment were much higher than those of the μ-LEDs without diluted HF chemical treatment. Even the smallest mesa size of the μ-LEDs with diluted HF treatment showed higher EQE values than the largest mesa size of the μ-LEDs without diluted HF treatment within all ranges of the operating current.

Further investigation into the influence of diluted HF chemical solution treatment on the optical properties was investigated. The enhanced ratios of EQE through diluted HF treatment were significantly different according to the injection current, as shown in Fig. 3B. The enhanced EQE ratios of the μ-LEDs with mesa sizes of 12, 16, and 19 μm were 35.48%, 26.11%, and 20.66% at an injection current of 15 μA and 12.86%, 8.98%, and 9.63% at an injection current of 100 μA, respectively. Compared with the overall enhanced EQE ratio in the region where it is nearly constant above the injection current of 50 μA, the improved EQE ratio of the low current range was much higher than that of the high current range. According to the injection current range, these transitions of the EQE ratio can be understood by considering the various contributors, such as improving the light extraction and decreasing the surface recombination. As shown in Fig. 2B, the geometry of the sidewalls is non-linear after the diluted HF chemical treatment. The surface area of the sidewalls near the active region is expected to expand further because of the selective etching of the AlInP layers. Particularly with the diluted HF chemical treatment, the active region’s calculated sidewall surface area (≈ 135 μm^2^) is expanded by more than 40% from the non-treatment case (≈ 95 μm^2^). In the case of a smaller LED size, the light emission from the sidewalls cannot be neglected owing to the large perimeter-to-area ratio^18^. The expansion of the sidewall surface area of the active region is desirable because it can help the photons generated from the active layers escape into the air by providing more pathways. Choi et al. investigated a similar study on the high surface area extraction provided by the sidewall-dependent performance^19^. Therefore, the nearly constant increase in the EQE ratio at a high injection current range can be explained by the improved sidewall extraction caused by the expansion of the surface area. However, we believe that the phenomenon of a low current range is closely associated with the suppression of the surface recombination, which is discussed in the following section.

**Figure 4:**
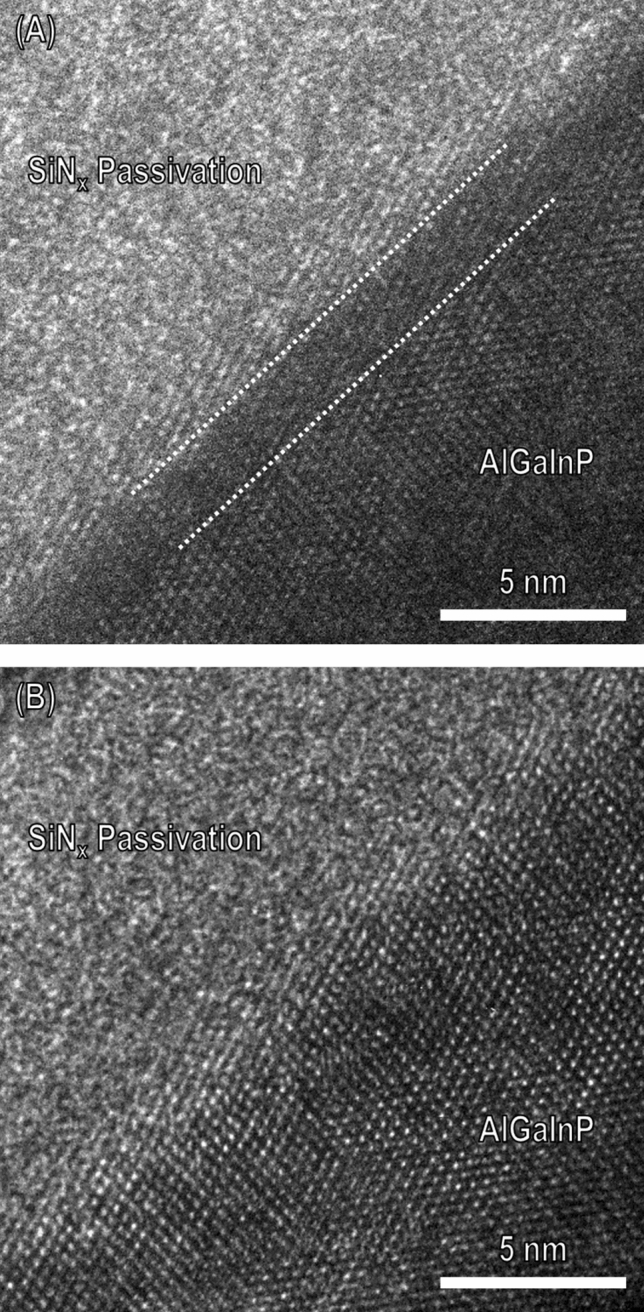
HR-TEM image of mesa sidewalls between passivation and active layers: (**A**) before diluted HF solution treatment and (**B**) after diluted HF (4 min) treatment.

The sidewalls of the LED can be liable to suffer plasma damage during the mesa etching process because energetic ion bombardment during the dry etching procedure can induce physical damage to the plasma-exposed surfaces^20–22^. A smaller LED size causes more surface damage owing to the large perimeter-to-area ratio^13,15^. Consequently, we expect that this will lead to a degradation of the LED performance. We investigated a TEM analysis of the surface region of the microstructure of the sidewall to recognize the plasma-induced damage. Figure 4A,B show HR-TEM images of the mesa slant sidewall interface between the active region and SiN passivation layer in the fabricated $\upmu$-LEDs. The AlGaInP of the active layer in the sidewalls shows a single crystalline atomic arrangement regardless of the chemical treatment, presumably owing to the lattice matching epitaxy. However, the HR-TEM images clearly show a significant difference in the crystal damage to the edge region of the sidewalls. The physical defects, such as a lattice disorder, were observed from the edge surface of the sidewalls with approximately a 20 Å thickness for the non-treatment case, as shown in Fig. 4A. This type of surface defect is consistent with the results discussed elsewhere. Lee et al. reported that physical defects remaining on the surface are strongly related to the plasma environment^23^. Pearton published a review of the ion-induced surface damage of III–V compound semiconductor materials^24^. The results of Fig. 4A also have a similar lattice morphology as described in previous studies after the ion bombardment process. By contrast, in the case of the diluted HF solution treatment, as shown in Fig. 4B, the edge surface of the sidewalls did not show any vestiges of crystal damage. We believe that the crystal damage in the region on the sidewall surface was shallowly etched through diluted HF treatment. The plasma-induced damage on the surface of the sidewall may be recovered by chemical treatment. This effect of the surface recovery can suppress the non-radiative recombination at the surfaces.

**Figure 5:**
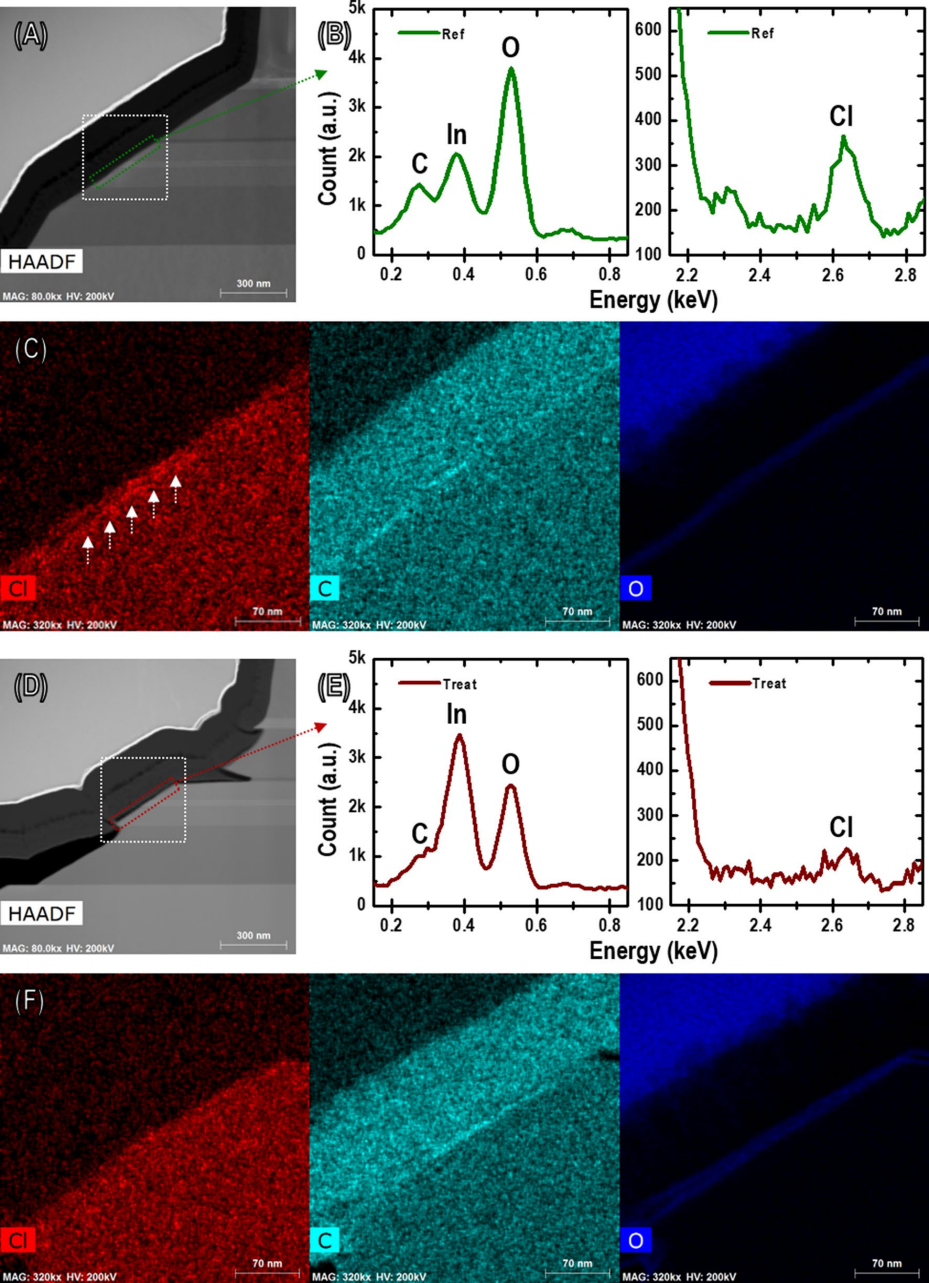
STEM-EDX analysis of mesa sidewalls (white box indicates mapping area in (**A**) and (**D**)). EDX spectra on mesa sidewalls (**B**) before diluted HF solution treatment and (**E**) after diluted HF (4 min) treatment; *a.u.* arbitrary units. EDX elemental (C, O, and Cl) mapping for mesa sidewalls (**C**) before diluted HF solution treatment and (**F**) after diluted HF (4 min) treatment.

A further compositional analysis was conducted to identify the residual elements of plasma gas on the sidewall surface by using energy-dispersive X-ray (EDX) spectroscopy. Figure 5A,D show low-magnification HAADF STEM images of the mesa sidewall region. Figure 5B,C,E,F show the STEM-EDX results of the slant sidewall surface. As shown in Fig. 5C,F, carbon and oxygen were observed along the slant sidewall surface in the case of both non-treated and diluted HF samples. The contamination of carbon and oxygen can also be played with surface defects, which cannot be unrelated to residual elements of the plasma gases. Another foreign element, chlorine (Cl), was clearly detected on the surface and was presumed to be the residual gas element of plasma in the non-treated case, as shown in the spectrum and EDX map of Fig. 5B,C. However, in the case of diluted HF treatment, the Cl element cannot be observed in the EDX map, and only a small Cl peak can be observed in the spectrum, as shown in Fig. 5E,F. The calculated Cl/In ratio in the non-treatment and diluted HF treatment cases were 4.71% and 1.61%, respectively. The incorporation of reactive Cl during ICP can explain the interface of the sidewalls. Afterward, reactive products of the etching, such as AlCl_3_, GaCl_3_, or InCl_3,_ may exist on the sidewall surface^25^, resulting in a deficient surface of the group-III species owing to the removal of group-III forming reactive products. Reactive Cl may initiate changes in the sidewall surface composition and lead to point defects or trap sites^25,26^. The traps play a role as a source of impurities or native defects. These undesirable surface states can act as non-radiative recombination centers. Several LED research approaches to improve the sidewall surface from plasma damage are still underway owing to their high efficiency^21,27^. However, plasma gases-related elements were not observed from the surface in the diluted HF treatment sample. All TEM results in our experiments clearly indicate the surface recovery effect of chemical treatment to suppress surface defects such as a lattice disorder and the incorporation of impurities. All types of surface defects, such as lattice distortion and foreign atoms, are the most common cause of an SRH non-radiative recombination to generate different energy levels^13,28^. The SRH non-radiative recombination at the surface caused by defects is one reason for the decreased LED efficiency, particularly at a low current density^17^. In addition, the efficiency of red LEDs using AlGaInP is more vulnerable to surface recombination at a low current density than InGaN-based LEDs owing to the minority carrier diffusion lengths related to a high SRV^14,29^. The recovery of surface damage can explain the high enhancement of EQE within the low-current region in Fig. 3B. The area of lattice distortion and residual impurities of plasma gases on the sidewall surface were eliminated by the diluted HF treatment procedure, as shown in Figs. 4 and 5. The surface of the recovered sidewalls by chemical treatment may improve their internal quantum efficiency by preventing a non-radiative recombination. This influence of surface recovery makes it even more efficient at low current densities because the surface defect sites became saturated when the carriers had elevated current densities. As a result, the improvement in the influence by reducing the surface combination decreases, and the effect of light extraction is more dominant in enhancing their EQE improvement. This can also explain the different behavior of the enhanced ratio of the EQE within the low and high current range in Fig. 3B. We therefore believe that our experimental results of improved EQE using diluted HF chemical solution treatment are related to combining both the increasing light extraction and suppressing the surface recombination.

## Conclusion

We have investigated and demonstrated the effect of diluted HF chemical solution treatment on the EQE of AlGaInP based $\upmu$-LEDs. The enhanced ratios of EQEs at high and low current ranges were measured as 35.48% and 12.86%, respectively. The AlInP cladding layers of the epi-structure and the surface edge of the active layers were etched using a diluted HF solution. The nonlinear geometry of the mesa sidewalls caused by selective etching is related to improving the light extraction efficiency at high injection current ranges. The recovered sidewalls surface by diluted HF acid chemical solution from plasma damage contributed to preventing the surface recombination caused by surface defects. In effect, the enhancement of EQE was more noticeable under low injection current ranges. A TEM analysis revealed sidewall surface defects such as amorphization and contaminants caused by the dry etching process. We believe that the diluted HF chemical treatment can be an effective candidate to achieve highly efficient AlGaInP-based $\upmu$-LEDs.
